# Four weeks of meditation training improves sustained attention in community-dwelling older adults: a proof-of-concept randomized controlled trial

**DOI:** 10.3389/fragi.2024.1322705

**Published:** 2024-03-01

**Authors:** Sabrina D. Ford, Lindsay S. Nagamatsu

**Affiliations:** ^1^ Neuroscience, Schulich School of Medicine and Dentistry, Western University, London, ON, Canada; ^2^ School of Kinesiology, Western University, London, ON, Canada

**Keywords:** meditation, sustained attention, event-related potentials, aging, cognitive function

## Abstract

**Introduction:** With our rapidly expanding population of older adults, identifying effective intervention strategies to improve cognitive functioning is an increasing priority. This study sought to examine whether 4 weeks of thrice-weekly meditation training can improve attention in older adults, as well as whether such benefits may extend to other domains of cognition as well as mobility.

**Methods:** Forty-three participants (mean age 68 years) were randomized into either the focused attention meditation group or the music listening control group (Clinicaltrials.gov ID NCT03417635). Participants completed three 20-minute guided group sessions per week for four consecutive weeks. Our primary outcome measure was behavioural performance on the Sustained Attention to Response Task (SART). Secondary and tertiary outcome measures included event-related potentials (ERPs) during the SART task, measures of executive functioning, and measures of mobility.

**Results:** We found that meditation training significantly improved attention, as demonstrated by improved SART accuracy and changes in N2 ERP amplitude and latency.

**Discussion:** These findings suggest that meditation may lead to changes in attention and underlying cognitive processing in older adults, although a full-scale definitive trial is needed. Future research on the long-term benefits with real world applications is warranted.

## 1 Introduction

Attention is a ubiquitous part of our everyday experience, directing and focusing our cognitive resources to specific external (e.g., environmental stimuli) or internal (e.g., thoughts) information. Aging is associated with progressive decline in multiple different cognitive functions, especially those involving the frontal lobe, including attention (e.g., Nagamatsu et al., 2011; Zanto and Gazzaley, 2019). Yet, attention is a critical cognitive function for completing activities of daily living; indeed, impaired attention has negative implications for a host of behaviours including driving (Anstey et al., 2005) and mobility (Yogev-Seligmann et al., 2008).

There is substantial evidence for the role of attention in falls risk in older adults. A cross-sectional study in cognitively healthy older adults found that poorer performance on executive attention tasks was significantly associated with both single and recurrent falls (Holtzer et al., 2007). Previous research has also found that older adults with a history of falls have alterations in their ability to attend to both task-relevant (Nagamatsu et al., 2009) and task-irrelevant (Nagamatsu et al., 2013b) visual information. These studies align with a recent systematic review that found that impaired attention is associated with falls risk (Marshall et al., 2023). Such results support the notion that attention is critical for balance and mobility, especially as we age (Althomali and Leat, 2018). Further, within the umbrella of attention, sustained attention – our ability to sustain our attention on a task for an extended period of time, may be of particular relevance. For example, older adults with a history of falls were found to spend more time mind-wandering and perform more poorly on a sustained attention task compared to those without a history of falls (Nagamatsu et al., 2013a).

Given the importance of attention in daily life combined with age-related declines, it is imperative that we find strategies to improve attention in older adults. One way to potentially improve sustained attention is through meditation. Meditation is the practice of bringing awareness to the present moment while attending to either one’s breath or a specific thought. While there are several different types of meditation, our study specifically employed focused attention (FA) meditation, where the practitioner focuses on the sensation of breathing while letting any thoughts or emotions drift through their awareness but not attend to them. A recent meta-analysis of mindfulness randomized controlled trials (RCTs) (*n* = 30) found that meditation significantly improves attention, particularly in healthy older adults without evidence of cognitive decline, and that the results were irrespective of key potential moderators, including age, intervention length, or gender (Mirabito and Verhaeghen, 2023). More specific for our study and the relationship with falls, there is mounting evidence suggesting that meditation can improve sustained attention (MacLean et al., 2010; Izzetoglu et al., 2020). For example, older adults with meditation experience were shown to have better sustained attention compared to age-matched non-meditators (Sperduti et al., 2016).

Even with an accumulation of evidence, there remain gaps in our knowledge of how meditation impacts cognition. Importantly, previous studies have been limited by their: 1) reliance on behavioural measures of attention which do not allow us to delve into the potential underlying neural changes that may be responsible for such changes, and 2) lack of real-world measures to gauge whether laboratory changes in cognitive functions translate to functions that rely on such processes, such as mobility. Therefore, to examine whether meditation can serve as an intervention strategy to improve sustained attention in older adults, we conducted a proof-of-concept study examining the effects of a four-week meditation intervention compared to a music listening control group. In addition to behavioural measures of sustained attention, we also used event-related potentials (ERPs) during task performance to assess electrophysiological responses in the brain. Importantly, inclusion of ERPs in our study to assess the underlying neural processing of sustained attention extends previous studies that have focused exclusively on behavioural assessments of attention; this will further our understanding of how meditation impacts attention by providing insight into changes at the neurophysiological level. To assess the effects of meditation training on other aspects of cognitive and physical functioning, we also included measures of executive function, mobility, and mood.

We hypothesized that 4 weeks of meditation training would lead to improvements in sustained attention compared to a music listening control group. Our control group was chosen as an active control to ensure that influences of socialization and participation in a group intervention were controlled for outside of the meditation training itself. The length of intervention was based on previous studies that have reported medium to large effect sizes for improving attention ranging from as little as four meditation sessions (Zeidan et al., 2010) to 2 months of training (Malinowski et al., 2017). In addition, the meta-analysis by Mirabito and Vergaeghen. (2023) examined studies with intervention lengths ranging from 2–96 weeks and found that intervention length was not a significant moderator contributing to the effects of meditation on cognition. Thus, 4 weeks was used within this study, although we acknowledge that studies with longer intervention periods and follow-up assessments would allow us to better understand the long-term effects of meditation training in older adults.

This proof-of-concept study aims to provide preliminary evidence for the above hypothesis and shed light on whether meditation as an intervention has the potential to improve sustained attention in healthy community-dwelling older adults. Given the high importance of sustained attention in everyday tasks, this intervention would provide an accessible strategy for older adults to improve a core cognitive function that may translate to better functional outcomes with age.

## 2 Methods

### 2.1 Study Design

We conducted a four-week single-blinded randomized controlled trial (RCT). Our study protocol has been published (Nagamatsu and Ford, 2019) and the trial was registered (Clinicaltrials.gov ID NCT03417635). The study was approved by the Health Sciences Research Ethics Board (HSREB) at Western University. The study took place from January to August 2019. Summary data are available upon request to the corresponding author.

#### 2.1.1 Changes to study protocol

As per our published protocol (Nagamatsu and Ford, 2019), we aimed to recruit a total of 60 participants (30 per group). We estimated that with an alpha level of 0.05, this sample size would provide a power of 0.81 to detect between-group differences post-intervention for our primary outcome measure. Due to the timing of our study which started in 2019 and led into multi-year university shutdowns that resulted from the COVID-19 pandemic, we terminated our proof-of concept RCT prior to obtaining our full sample size. Consequently, we adjusted our significance level from our proposed 0.05 to 0.10 to maintain our intended power (0.77 vs. 0.81) to detect intervention-related changes. Since this is a proof-of-concept study, rather than a full-scale definitive trial, having a more liberal threshold is important to be able to have a preliminary assessment of potential effects prior to investing in a full-scale trial. Covariates were not included in our analysis as we did not see any preliminary relationship between our variables of interest and potential covariates; this is in line with the meta-analysis by Mirabito and Verhaeghen. (2023) who showed that such demographic factors (age, sex, etc.) did not moderate the effects of meditation on cognition. For the intention-to-treat analysis, we included all participants where data was collected at both time points (baseline and endpoint) as per clinical trial recommendations (Altman, 2001). For those participants that dropped out after baseline assessments and did not complete any training sessions, they were not included in the analysis because we did not have endpoint data from them (they declined to come back for the endpoint assessment). All participants with data at both time points were included, regardless of how many meditation/control sessions they attended or whether data was not collected for one or more variable (e.g., left-handed participants who did not complete the EEG were still included in the other cognitive and mobility analyses).

### 2.2 Participants

Older adults living in London, Ontario, Canada were eligible for the study if they met the following inclusion criteria: 1) living independently, 2) complete high school, 3) able to read and write in English, 4) able to walk independently, 5) scored ≥6/8 on the Instrumental Activities of Daily Living (IADL) scale, and 6) scored >24/30 on the Mini-Mental Status Examination. Participants were excluded from the study if they met any of the following exclusion criteria: 1) diagnosed with a neurodegenerative disease, 2) diagnosed with cognitive impairment (e.g., mild cognitive impairment, MCI), 3) diagnosed with a psychiatric condition (e.g., depression), 4) sustained a concussion in the past 12 months, 5) have had a stroke, 6) have musculoskeletal or joint disease, 7) experience dizziness or a loss of balance, 8) have visual, auditory, or somatosensory impairment that is uncorrected, or 9) have a recent history (past 2 years) of meditation practice or include a meditation component in their religious practice.

Participants were recruited from within the community through various groups such as an Alumni group at the local university and local senior’s community centers. Flyers were distributed at the centers and the study was presented at several community meetings with the aforementioned groups. Advertisements were also distributed online and in a local newspaper. As per our published protocol, we estimated that to detect between-group differences with an alpha set at 0.05 and assuming a medium effect size, we would require a total of 52 participants in our study to obtain a power of 0.8075. Notably because this is a proof-of-concept study meant to examine the potential for this training protocol to have an effect on attention, we used a liberal threshold of *p* ≤ 0.10 and had a smaller sample size than was required for a full-scale trial.

### 2.3 Procedure

Participants who were interested in the study contacted the lab via email or phone, where they were screened for study inclusion. If they met the inclusion criteria, they were invited into the lab for two separate baseline assessment sessions. The first baseline assessment session included going through the study details, obtaining informed consent, and collecting all demographic, cognitive, mobility, and psychological measures. During the second baseline assessment session participants completed the SART task while we recorded EEG. The sessions were on average 10 days apart, with a range of 1–42 days. Upon completion of the baseline assessments, participants were randomly assigned to one of two groups: the meditation (experimental) group or the music (control) group. Randomization was completed using a random sequence generator (randomization.com) with random block sizes of 2, 4, and 6. The sequence was held remotely by the Principal Investigator and group allocation was only revealed after baseline assessments were complete. After completing the four-week intervention, participants returned for the same two assessment sessions (Session 1: cognitive, mobility, and psychological measures; Session 2: SART task and EEG). All baseline and follow-up assessments were completed by an assessor who was blinded to group allocation.

### 2.4 Intervention

The intervention was run in-person at Western University. All sessions were led by the lead author who had 1 year of experience as a mediation instructor and 4 years of personal meditation experience. Each session was 20 min in duration and participants completed three sessions per week for four consecutive weeks (a total of 12 sessions). Attendance was monitored and recorded throughout the study. Sessions were completed in groups of one to five participants, with an average of three participants per group.

Participants were encouraged to practice similar style meditations outside of the regularly scheduled sessions, and those in the music group were given a copy of the music track (i.e., CD or USB) to listen to on their own time. Participants were asked to meditate or listen to the music track in a way that mimics the activities done in the groups.

#### 2.4.1 Meditation (experimental) group

Participants were given the option to sit in a chair with their feet on the floor or lie down on the floor with a yoga mat. The option to keep eyes closed throughout the meditation or have them open and unfocused on a spot on the floor was given each session. All meditations were focused on the breath, meaning that participants were asked to maintain their attention on their breathing and were periodically reminded to re-focus their attention back to their breath when their attention wandered or they became distracted. Each meditation began with participants taking a moment to settle into their position and then to bring their attention to the rate and depth of their breathing without trying to change their breath. Attention was then brought to the participants’ bodies where they would explore if they had any tension in their stomach, their shoulders, their face, or anywhere else. Participants were then instructed to use their exhales to let go of that tension or stress that they brought with them. Three different techniques were introduced throughout the 12 sessions and participants were given the option to use the techniques or not depending on their preference. The three techniques were: 1) focusing on the sensation of the breath going in and out through their nose, 2) imagining a string pulling at the top of their head with each inhale and their shoulders relaxing with each exhale, and 3) counting each breath up to 10 and then restarting back at one. Participants were taught one technique per meditation for the first week and were then given the choice to choose one technique or use their own for the remainder of the sessions. As the sessions progressed, the amount of silence increased and the reminders to re-focus attention on the breath if the mind wandered decreased. At the end of the 20-minute meditation, participants were asked to focus their attention back to their breath, then back to the room around them, and then to slowly open their eyes.

#### 2.4.2 Music (control) group

As with the meditation group, participants in the music group were given the option to sit in a chair with their feet on the floor or laying down on a yoga mat. An instrumental jazz track was played during these sessions, and participants were asked to close or relax their eyes and let their mind wander while listening to the music.

### 2.5 Descriptive Measures

General demographic information, including age, sex, and education were ascertained via questionnaire. Participants completed the Montreal Cognitive Assessment (MoCA) and Mini-Mental Status Examination (MMSE) to assess global cognition. The Instrumental Activities of Daily Living (IADL) scale was used to assess the ability to complete daily activities such as shopping, using transportation, and preparing meals. The Geriatric Depression Scale (GDS) was used to screen for depression. The Functional Comorbidity Index was used to quantify number of comorbidities. Finally, participants reported the number of falls they experienced over the past 6 months via questionnaire.

### 2.6 Primary outcome measure

Our primary outcome measure was behavioural performance on the sustained attention to response task (SART). The SART is a go/no-go task which requires participants to attend and manually respond to visual stimuli and withhold responses to an infrequent target. Success on the SART requires prolonged, sustained attention to the stimuli. Previous research has found that performance on the SART can be improved after meditation training in young adults (Rahl et al., 2017).

**FIGURE 1:**
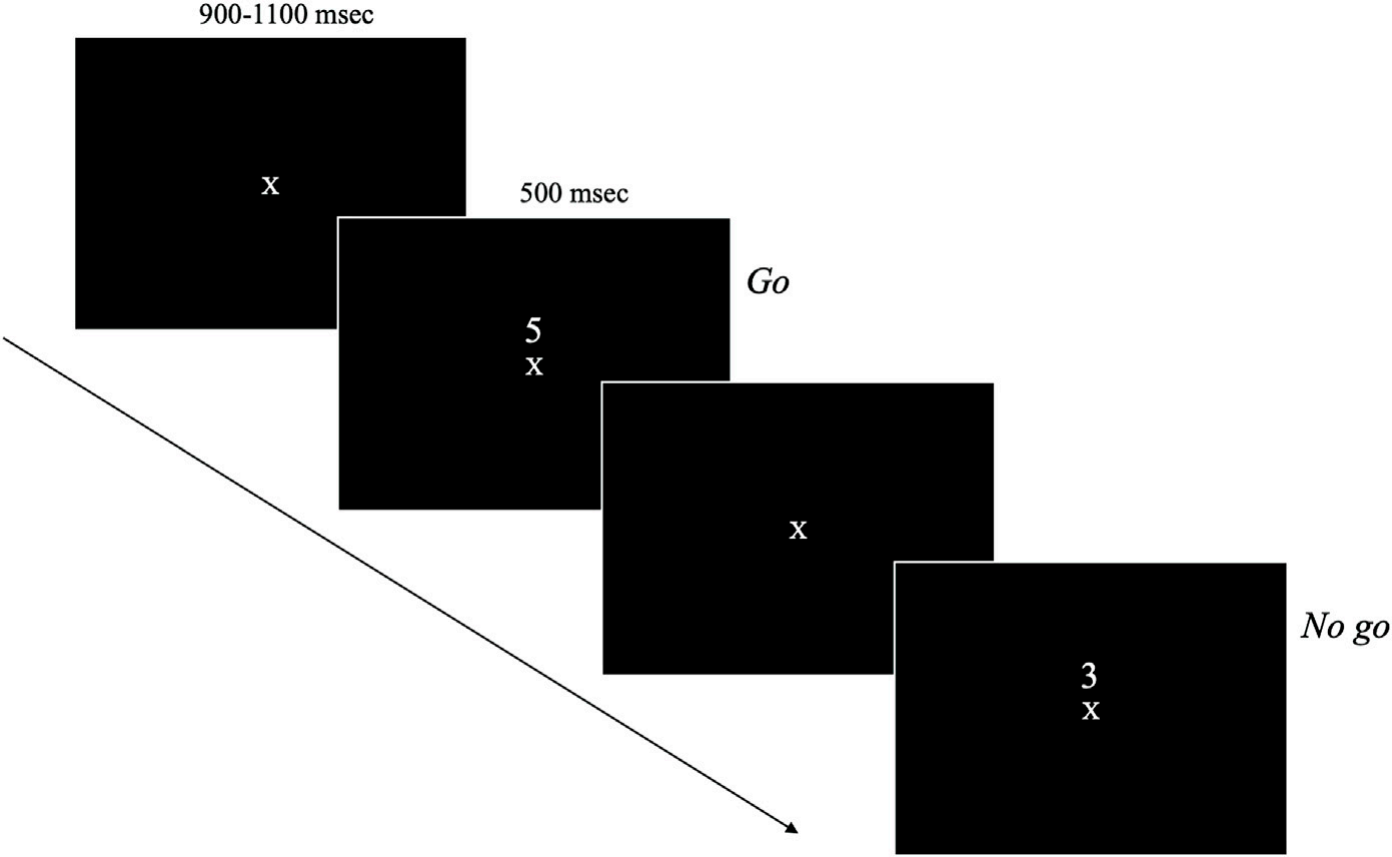
SART stimulus presentation and timing.

Stimulus presentation and timing is illustrated in Figure 1. Participants were seated 34-inches from a 25-inch monitor mounted 43-inches from the ground. On the monitor, a random sequence of single digits (i.e., the numbers 0–9) were presented one at a time in the centre of the screen for 500 msec with a 900–1,100 msec randomly jittered interstimulus interval. Participants were instructed to respond by pressing a button with their right index finger on a gamepad controller each time a digit was presented with the exception of the target (the number “3”) which they had to withhold their response. Participants had until the next stimuli was presented to respond to the current stimuli. Participants were instructed to respond as quickly and accurately as possible. Each block was randomly assigned to present either 5 or 7 targets. Each participant completed 14 blocks with 60 stimuli in each, which took approximately 35 min in total to complete, including a mandatory break at the half-way mark. Participants were also given the opportunity to take a break after each block. Left-handed participants (*n* = 2 in the meditation group and *n* = 1 in the music group) did not complete the SART task.

### 2.7 Secondary outcome measures

During performance of the SART task we recorded event-related potentials (ERPs) to examine underlying attentional and cognitive processing. For the ERPs, we focused on the N2 and P3 components, as visual go/no-go tasks typically elicit a frontally mediated inhibitory response that can be measured via a negative deflecting component which reaches peak amplitude at electrode site Fz with a latency of 150–400 msec (termed the “No-go-N2”) and a positive deflecting component which reaches maximum amplitude at Fz and Cz at a latency of 300–500 msec (termed the “No-go-P3”) (Falkenstein et al., 1999).

We recorded electroencephalograms (EEGs) from 64 active electrodes using an actiCHamp system from BrainVision Brain Products GmbH (Herrsching, Germany). The electrodes were placed according to the 10–20 system, with the ground electrode placed at Fpz and the reference channel at AFz. Data was recorded with BrainVision Pycorder with a low pass filter of 0.05 Hz and a sampling rate of 500 samples-per-second. Impedances were kept under 20 kW. Electrodes were also placed on the mastoids for offline re-referencing and below and beside the eyes to record vertical and horizontal electrooculograms (EOGs) to detect eye movements and blinks.

Analysis of the EEG data was done using ERPLab (https://erpinfo.org/erplab), an open source Matlab package. Data was first re-referenced to the average of the two mastoids. Events were grouped into bins according to trial type, with only trials with a correct response included. Bin-based epochs were then extracted, from −200msec pre-stimulus to +1000msec post-stimulus with a pre-stimulus baseline correction. Artifact rejection was conducted using moving window peak to peak based on EOG channels. Averaged ERPs were then computed. Next, data was low- and high-pass filtered using the Butterworth option in ERPlab, at 30 and 0.1 Hz, respectively.

Based on the extant literature, we focused on the N2 and P3 ERP components (Falkenstein et al., 1999; van Gaal et al., 2008; Zordan et al., 2008). For each time point (baseline and trial completion), each trial type (go and no-go), and each group (meditation and music) we extracted the peak latency and mean amplitude based on the grand-averaged waveforms. Peak latency was defined as the timepoint with the highest amplitude in the 200–400 msec range for the N2 and the 400–600 msec range for the P3. Mean amplitude was defined as the average amplitude in the 40msec window (±20 msec) of the peak latency. Both peak latency and mean amplitude were extracted for midline electrode sites (Fz, Cz, Pz, Oz).

### 2.8 Tertiary outcome measures

#### 2.8.1 Executive functions

Participants complete three separate paper-based tests of executive function based on Miyake’s proposed domains of executive function (Miyake et al., 2000). Working memory was tested using the Digit span test where participants had to remember increasingly long sequences of digits in both the forward direction (Digits Forwards) and the reverse direction (Digits Backwards). Scores were calculated as the difference between number of digits correctly remembered forward and backward. Set-shifting was tested using the Trail Making Test where participants had to connect in sequence encircled numbers (Part A) and numbers and letters alternating (Part B). Scores were calculated as the difference in time to connect all circles in Part B and Part A. Response inhibition was tested using the Stroop Colour Word Test where participants had to verbalize the colour of ink that x’s were written in (neutral condition, Part B) or that colour words were written in (incongruent condition, Part C). Scores were calculated as the difference in time to complete Part C and Part B.

#### 2.8.2 Mobility-related measures

Self-reported confidence in one’s balance was ascertained using the Activities Balance Confidence (ABC) scale (Powell and Myers, 1995). Participants rate their confidence to complete 16 separate tasks from 0 (not confident at all) to 100 (completely confident). The average of the 16 items is averaged for a total score out of 100. Mobility was tested using the Timed Up and Go (TUG) test (Podsiadlo and Richardson, 1991), where participants stand up from a seated position, walk 3 m, turn, return to their seat and sit back down. The average time to complete two trials was recorded in seconds. Physical performance was assessed using the Short Physical Performance Battery (SPPB) (Guralnik et al., 1994) where participants complete a series of standing tasks (side-by-side, tandem, semi-tandem), a four-meter walk, and five chair stands. Each is scored out of a total four points, for a total score out of 12.

#### 2.8.3 Mood and attention

Mood was assessed using the Depression, Anxiety, and Stress Scale (DASS) (Lovibond and Lovibond, 1995), a 21-item scale that assesses mood. Participants rate how much each of the 21 items apply to them on a four-point Likert scale. The items are divided into three subscales which measure depression, anxiety, and stress, with higher scores indicating higher levels of those moods.

The Cognitive Failures Questionnaire (CFQ) (Broadbent et al., 1982) was used to measure the frequency that participants experience failures in attention in everyday life. This self-report 25-item questionnaire asks participants to rate on a five-point Likert scale how much each item pertains to them. Examples include “Do you find you forget people’s names?”. Higher scores indicate more cognitive failures.

### 2.9 Statistical analysis

Baseline characteristics were examined between groups using independent samples t-tests for continuous data and chi-squared tests for nominal data. For all measures of interest (primary, secondary, and tertiary outcomes) we conducted repeated measures ANOVAs with factors of time (pre-vs. post-intervention) and group (meditation vs. control). For the ERP analysis, we also included a factor of electrode site (Fz, Cz, Pz, Oz), however for the purposes of this paper, any significant main effects of electrode site are not reported below because they are tangential to the purpose of this study (i.e., examining effects over time and between groups). For analyses where the assumption of sphericity was not met, Greenhouse-Geisser values are reported with adjusted degrees of freedom. Notably because this is a proof-of-concept study, *p* values were set at ≤ 0.10 to enable us to assess potential effects of meditation on attention, executive function, and mobility in a smaller sample. To better characterize intervention changes, we also report effect sizes using partial eta squared (η_p_^2^), where we interpret 0.01 to indicate a small effect, 0.06 a medium effect, and 0.14 a large effect. All data was analyzed in SPSS (Version 27 for Mac).

## 3 Results

### 3.1 Descriptive Measures

**FIGURE 2:**
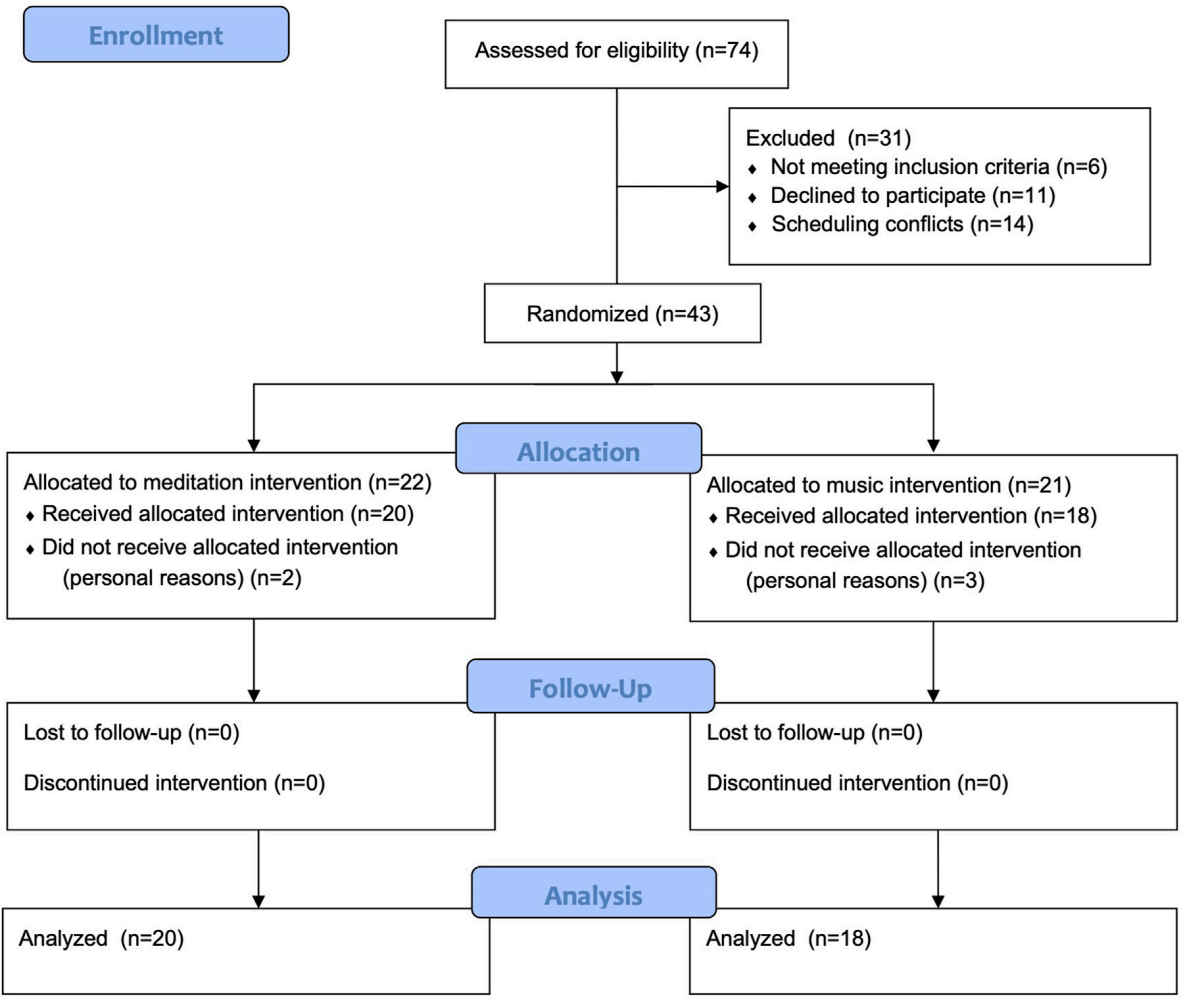
CONSORT flow diagram.

Descriptive data is presented in Table 1 and participant flow through the study is presented in Figure 2 (CONSORT flow diagram). Forty-three participants were randomized, with 22 in the meditation intervention and 21 in the music intervention. Five participants dropped out of the study prior to starting the intervention (two in the meditation intervention and three in the music group) leaving a final sample size of 38. Overall, participants were an average age of 68.53 (SD = 5.42) years old (range 57–80 years), had high cognitive functioning as assessed by the MMSE, and were able to function independently as measured by the IADLs. There were no significant differences between groups at baseline. All participants completed all 12 sessions of training.

**TABLE 1:** Baseline demographic characteristics of the sample.

	Music group (*n* = 18)	Meditation group (*n* = 20)	Difference between groups (*p*-value)
Age, years	68.06 (4.92)	68.95 (5.93)	0.62
Female, no. (%)	12 (66.67)	11 (55.00)	0.46
Education, no. (%)			0.87
High school diploma	0 (0.00)	2 (10.00)	
Trade certificate	1 (5.60)	6 (30.00)	
Some college	7 (38.90)	0 (0.00)	
Bachelor’s degree	6 (33.3)	11 (55.00)	
Graduate degree	4 (22.2)	1 (5.00)	
MoCA^a^	24.78 (2.24)	25.40 (2.74)	0.45
MMSE^b^	28.30 (1.34)	27.94 (1.39)	0.43
IADL^c^	8 (0.00)	7.95 (0.22)	0.35
GDS^d^	1.28 (1.45)	1.00 (1.12)	0.51
FCI^e^	1.78 (1.67)	1.35 (1.14)	0.36
Number of falls in the past 6 months	0.39 (0.85)	0.32 (0.58)	0.76

Note. Statistics represent the mean (SD) unless otherwise indicated.

^a^ Montreal Cognitive Assessment, scored out of 30.

^b^ Mini-Mental Status Examination, scored out of 30.

^c^ Instrumental Activities of Daily Living scale, scored out of 8.

^d^ Geriatric Depression Scale, scored out of 15.

^e^ Functional Comorbidity Index, scored out of 18.

*p < 0.100.

### 3.2 Primary Outcome Measure

Behavioural data is presented in Table 2. Full repeated measures ANOVA tables are also presented in Supplementary Table S5 (Supplementary Material). Three participants were excluded from the analysis for behavioural data (data did not save properly, *n* = 1 for meditation, *n* = 1 for music; one participant from the meditation group declined to complete the SART at endpoint for personal reasons).

**TABLE 2:** Behavioural performance on the SART.

	Music group (*n* = 16)		Meditation group (*n* = 16)	
	Baseline	Endpoint	Baseline	Endpoint
Go trials				
Accuracy (%)	99.79 (0.23)	99.09 (2.11)	98.58 (2.32)	99.66 (0.47)*
Reaction time (msec)	366.06 (43.95)	357.84 (65.62)	400.72 (68.49)	423.19 (99.87)
No-go trials				
Accuracy (%)	92.20 (7.92)	93.98 (6.28)*	92.27 (5.39)	96.67 (2.75)*

*p < 0.100.

For go trials, participants in the meditation group significantly improved their accuracy post-intervention compared to the control group, as evidenced by a significant time x group interaction, F(1,30) = 5.599, *p* = 0.025, η_p_^2^ = 0.157. There were no differences over time or between groups for reaction times, all *p*’s > 0.169.

For no-go trials, participants in both groups improved their accuracy post-intervention, as shown by a main effect of time, F(1,30) = 15.752, *p* < 0.001, η_p_^2^ = 0.344. However, participants in the meditation group improved significantly more than those in the control group, as demonstrated by a significant time x group interaction, F(1,30) = 2.838, *p* = 0.102, η_p_^2^ = 0.086.

Examining correlations in behavioural performance, slower reaction time to go trials was positively correlated with correctly withholding responses on no-go trials, r(34) = 0.501, *p* = 0.001, demonstrating that adopting a slower, more careful strategy was associated with more accurate performance.

### 3.3 Secondary outcome measures

#### 3.3.1 N2 ERP component

**FIGURE 3:**
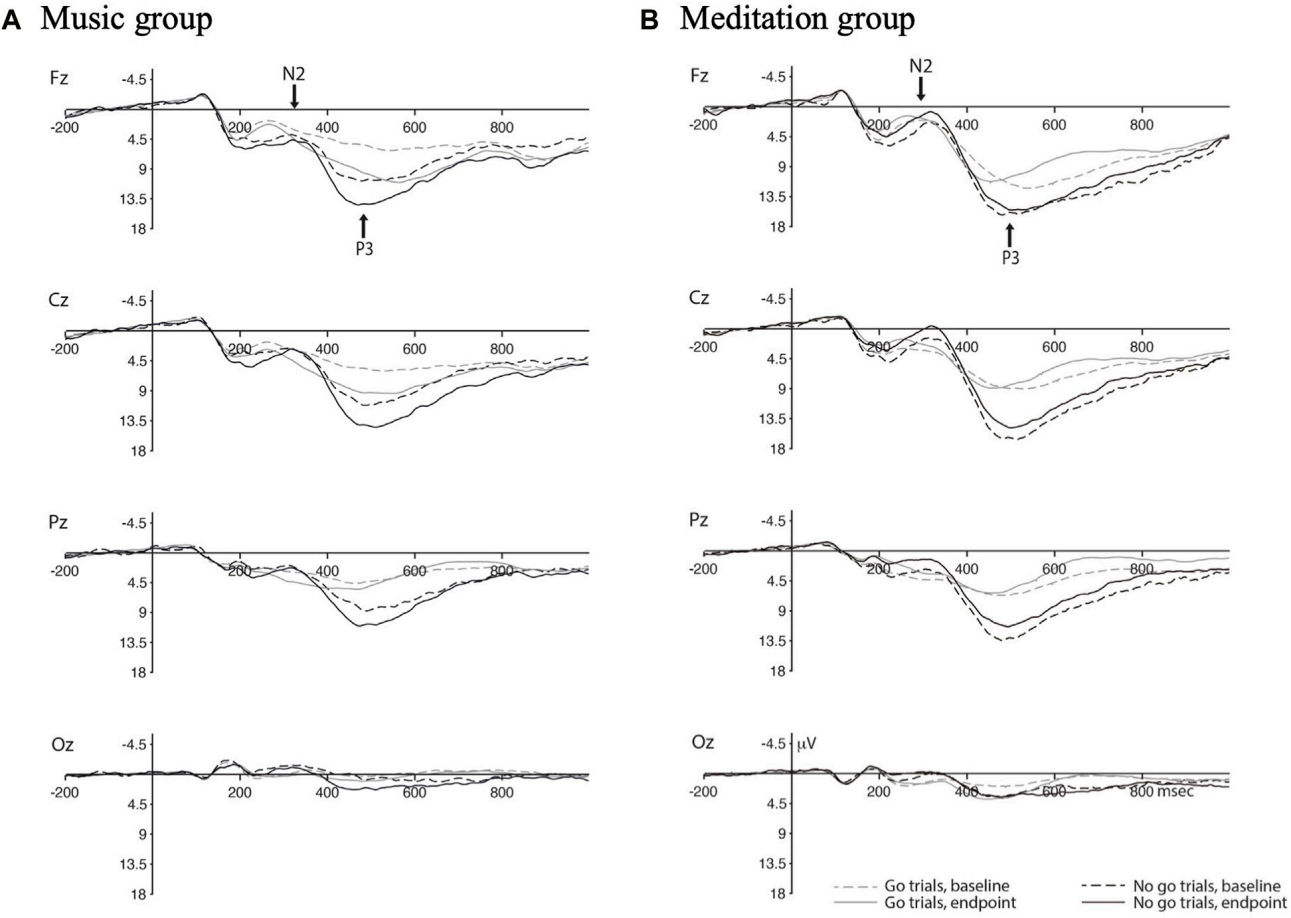
ERP waveforms for the Music group **(A)** and Meditation froup **(B)** with separate waves for go vs. no-go trials at baseline and endpoint.

ERP waveforms are presented in Figure 3 and mean amplitudes and peak latencies are presented in Table 3. Three participants were excluded from the analysis for ERP data (ERP data was excessively noisy, *n* = 1 for meditation, *n* = 1 for music; one participant from the meditation group refused to complete the SART at endpoint).

**TABLE 3:** Mean amplitudes and peak Latencies for the N2 and P3 ERP components.

				Music group (*n* = 17)		Meditation group (*n* = 15)	
				Baseline	Endpoint	Baseline	Endpoint
N2 component	Mean amplitude^a^	Go trials	Fz	1.94 (2.87)	2.56 (4.05)	2.18 (3.43)	1.65 (3.82)
			Cz	2.07 (2.55)	2.92 (3.47)	3.10 (2.74)	1.94 (3.23)
			Pz	2.64 (2.60)	3.23 (3.35)	4.30 (2.86)	3.24 (3.67)
			Oz	−0.12 (3.09)	0.26 (2.17)	1.11 (1.34)	1.45 (1.86)
		No-go trials	Fz	4.04 (4.71)	5.08 (7.32)	2.55 (4.01)	1.08 (4.03)
			Cz	2.70 (4.77)	3.41 (5.24)	1.58 (4.10)	0.11 (4.26)
			Pz	2.26 (2.97)	2.53 (4.91)	2.95 (2.88)	1.34 (3.76)
			Oz	−1.21 (3.90)	−0.90 (2.94)	0.08 (1.97)	−0.10 (3.05)
	Peak latency^b^	Go trials	Fz	280.24 (39.03)	278.00 (43.20)	280.40 (39.85)	291.20 (43.46)
			Cz	287.18 (41.16)	264.00 (47.33)	282.53 (49.43)	278.27 (49.44)
			Pz	289.76 (51.89)	267.41 (54.95)	300.27 (62.66)	297.47 (59.57)
			Oz	313.41 (65.35)	302.47 (59.28)	322.13 (56.71)	309.87 (67.43)
		No-go trials	Fz	303.53 (58.07)	302.35 (57.92)	314.13 (46.66)	308.40 (41.04)
			Cz	307.41 (58.66)	295.41 (52.53)	306.40 (51.07)	300.13 (48.99)
			Pz	280.00 (55.71)	298.47 (47.10)	296.67 (39.56)	296.00 (50.43)
			Oz	298.35 (49.64)	302.59 (50.78)	319.47 (55.08)	275.20 (55.11)
P3 component	Mean amplitude	Go trials	Fz	5.79 (6.62)	10.41 (9.41)	11.85 (13.21)	11.08 (13.01)
			Cz	5.90 (4.70)	9.29 (5.58)	8.92 (7.83)	8.78 (9.43)
			Pz	4.41 (3.58)	5.42 (4.91)	6.65 (5.51)	6.29 (6.47)
			Oz	0.30 (2.54)	0.97 (2.22)	1.88 (2.54)	3.81 (7.48)
		No-go trials	Fz	10.67 (6.80)	14.20 (9.99)	15.98 (4.36)	15.37 (10.32)
			Cz	11.01 (6.33)	14.31 (7.74)	16.45 (5.30)	14.69 (6.88)
			Pz	8.48 (6.12)	10.77 (5.37)	13.19 (4.79)	11.20 (5.72)
			Oz	0.61 (5.52)	2.14 (4.20)	3.43 (4.37)	3.41 (4.72)
	Peak latency	Go trials	Fz	506.94 (64.53)	521.53 (64.41)	513.87 (67.81)	468.00 (58.37)
			Cz	511.53 (63.97)	499.18 (61.32)	502.40 (56.40)	474.40 (46.98)
			Pz	488.24 (44.12)	470.82 (57.95)	475.60 (44.54)	460.53 (37.17)
			Oz	479.76 (50.33)	478.47 (57.46)	481.60 (59.02)	455.73 (37.06)
		No-go trials	Fz	512.59 (56.41)	502.47 (53.34)	505.20 (46.40)	503.73 (53.65)
			Cz	502.94 (51.12)	505.18 (48.37)	500.00 (37.37)	500.27 (47.36)
			Pz	504.00 (56.40)	505.76 (39.32)	495.87 (40.09)	494.67 (45.35)
			Oz	508.94 (58.03)	503.41 (64.75)	488.53 (38.36)	493.73 (49.93)

^a^ Mean amplitudes measured in μV.

^b^ Peak latency measured in msec post-stimulus.

For go trials, the peak latency of the N2 ERP component shifted earlier post-intervention for both groups, as evidenced by a main effect of time, F(1,30) = 3.652, *p* = 0.066, η_p_^2^ = 0.109.

For no-go trials, the mean amplitude of the N2 ERP component increased (became more negative) in the meditation group post-intervention compared to the control group. This was demonstrated by a significant time x group interaction, F(1,30) = 3.221, *p* = 0.083, η_p_^2^ = 0.097. Similarly, the peak latency of the N2 ERP component shifted earlier in the meditation group post-intervention compared to the control group, with a significant time x group interaction, F(1,30) = 3.029, *p* = 0.092, η_p_^2^ = 0.092.

#### 3.3.2 P3 ERP component

For go trials, there was an overall increase in mean amplitude in the P3 ERP component post-intervention for both groups, as shown by a significant main effect of time, F(1,30) = 3.347, *p* = 0.077, η_p_^2^ = 0.100. These increases in amplitude were mostly being driven by the control group in the frontal electrode site, as demonstrated by a significant time x electrode x group interaction, F(1.472, 44.162) = 4.223, *p* = 0.031, η_p_^2^ = 0.123. There was also an overall decrease in peak latency of the P3 ERP component for both groups after the 4 week intervention with a significant main effect of time, F(1,30) = 3.123, *p* = 0.087, η_p_^2^ = 0.094.

For no-go trials, the P3 ERP component increased in mean amplitude post-intervention for the control group and decreased for the meditation group, as shown by a significant time x group intervention, F(1,30) = 3.583, *p* = 0.068, η_p_^2^ = 0.107.

### 3.4 Tertiary outcome measures

#### 3.4.1 Executive functions

Results from our tertiary outcomes are presented in Table 4. For working memory, the meditation group significantly improved their Digit span performance post-intervention compared to the control group, as evidenced by a significant time x group interaction, F(1,36) = 5.437, *p* = 0.025, η_p_^2^ = 0.131. There were no significant time, group, or time x group effects for set-shifting or response inhibition.

**TABLE 4:** Cognitive, mobility, and mood measures.

	Music group (*n* = 18)		Meditation group (*n* = 20)	
	Baseline	Endpoint	Baseline	Endpoint
Digit span (forwards – backwards)	1.44 (2.33)	1.89 (1.88)	1.90 (1.77)	0.70 (2.15)*
Trail making test (part B – part A)^a^	28.20 (21.71)	29.50 (16.41)	27.84 (15.88)	33.43 (21.44)
Stroop colour word test (part C – part B)	40.70 (17.93)	40.18 (15.42)	39.38 (22.15)	39.13 (20.39)
Activities balance confidence scale	94.40 (6.45)	96.01 (4.43)*	94.70 (3.64)	96.64 (3.01)*
Timed up and go test	9.12 (1.70)	8.33 (1.59)*	8.50 (1.49)	8.04 (1.57)*
Short physical performance battery^b^	10.24 (1.20)	10.56 (1.29)	10.56 (1.10)	10.65 (1.23)
Depression anxiety stress scale				
Depression	3.83 (4.18)	3.39 (3.58)	2.90 (4.23)	1.75 (2.43)
Anxiety	2.50 (2.18)	1.67 (2.28)*	5.00 (6.04)	2.55 (2.98)*
Stress	6.28 (3.85)	5.00 (4.58)*	7.20 (5.78)	4.70 (4.51)*
Cognitive failures questionnaire	31.67 (7.22)	32.67 (7.50)	35.75 (9.92)	31.75 (7.83)*

^a^ 1 person in meditation group did not complete Trail making at endpoint.

^b^ 1 person in music group and 2 people in meditation group did not complete SPPB, at baseline.

*p < 0.100.

#### 3.4.2 Mobility-related measures

Both groups improved their balance confidence post-intervention, as demonstrated by a significant main effect of time on the ABC Scale, F(1,36) = 6.640, *p* = 0.014, η_p_^2^ = 0.156. Similarly, both groups improved their mobility post-intervention, as assessed by the TUG, with a main effect of time, F(1,36) = 6.829, *p* = 0.013, η_p_^2^ = 0.159. Physical performance as assessed by the SPPB showed no changes over time for either group.

#### 3.4.3 Mood and attention

For the DASS, both groups significantly reduced their anxiety and stress post-intervention, as shown by significant main effects of time for both the anxiety and stress subscales, F(1,36) = 4.828, *p* = 0.035, η_p_^2^ = 0.118 and F(1,36) = 6.172, *p* = 0.018, η_p_^2^ = 0.146, respectively.

For the cognitive failures questionnaire, the meditation group reported fewer instances of absent-mindedness post-intervention compared to the control group, with a significant time x group interaction, F(1,36)=3.862, *p*=0.057, η_p_^2^=0.097.

## 4 Discussion

We conducted a proof-of-concept four-week meditation intervention to examine the effects of meditation training on attention and other cognitive and physical outcomes in older adults. To that end, we report that our intervention improved sustained attention, as measured by SART accuracy, which aligns with a previous meta-analysis that demonstrated consistent evidence that meditation training improves attention in older adults (Mirabito and Verhaeghen, 2023). However, extending previous literature, we also showed changes in N2 ERP component amplitude and latency, providing insight into the neural changes that correspond to changes in attentional performance. These findings will be discussed in context of relevant literature below.

First, our behavioural results indicate that the meditation group improved their accuracy for both go and no-go trials, reflecting better inhibition for correctly withholding responses during no-go trials. Whether or not this improvement in accuracy after meditation training reflects a speed-accuracy tradeoff remains unresolved. While we did not observe between-group differences in reaction time, we did find an overall significant correlation across all participants between reaction time and accuracy, such that slower reaction times were associated with higher accuracy. There are previous reports that experienced meditators (e.g., monks) and those that undergo meditation training have slower reaction times compared to control participants (Naranjo and Schmidt, 2012). However, there is also evidence that meditation can improve the efficiency of attentional processing without overt changes in reaction time (van den Hurk et al., 2010). We also note that a ceiling effect was observed for performance accuracy, particularly for go trials. While this may impact our ability to reliably detect differences between groups or timepoints, the pattern of results was similar for no-go trials but with greater variability in the data, thus increasing our confidence in the results. While future research is needed to shed light on the impact of meditation training on reaction time, results from this study suggest that meditation can improve sustained attention and inhibition at a behavioural level.

Second, focusing on the “no-go” ERP waveforms, we found that meditation training resulted in an increased amplitude and shorter latency for the N2 component and a smaller amplitude for the P3 component. The no-go N2 is thought to reflect inhibitory control, where participants with better inhibition (as exhibited by fewer false alarm rates in a go/no-go task) have a larger and earlier N2 component (Falkenstein et al., 1999). These results directly align with our findings of this exact pattern of results in our meditation group; that is, meditation training appears to have resulted in better inhibitory control as measured via ERPs. This corroborates reports that meditation training increases white matter connectivity with tracts connecting the anterior cingulate cortex (ACC), an area implicated in inhibitory control (Tang et al., 2010), with other brain structures. This also suggests that the observed behavioural effects were not exclusively due to a slower, more careful approach, but rather a change in executive processing. In contrast, the no-go P3 has been linked to motor inhibition (i.e., inhibiting the motor response required during SART-like tasks) (Smith et al., 2008). The discrepant results between the N2 and P3 in our study may reflect shifts in cognitive strategy and need to be further examined in future studies.

Third, we found that our meditation group significantly improved their working memory performance but not set-shifting or response inhibition. In a recent systematic review, Gallant. (2016) examined the effects of meditation training on the three subcomponents of executive function used in our own study (working memory, set-shifting, and response inhibition). From the 12 studies included in the review, response inhibition was found to have the most robust improvements after meditation training, followed by working memory, with the least amount of evidence for set-shifting. The fact that we did not see improvements in response inhibition is surprising given the corresponding improvements in the SART task which also involves inhibition; however, it is possible that our pen and paper version of the Stroop task was not sensitive enough to detect changes over time, or that meditation training impacts different domains of inhibition differently (language/verbal versus visual/motor). Future research into the effects of meditation on specific domains of executive function is warranted.

Finally, we found that both groups had reduced anxiety and stress post-intervention, which suggests that there may be multiple different ways to impact mood and mental health in this population. Our intervention and control groups had nearly identical experiences other than the meditation aspect, including time commitment, socialization opportunities, and an element of relaxation. Previous research has advocated for the critical role that socialization plays in wellbeing and mental health, especially as we age (Fiori et al., 2006; Newman and Zainal, 2020). However, we note that this result indicates that a mere reduction in anxiety and stress are not driving our results with respect to improved attention – that is, meditation has potential cognitive benefits above and beyond improvements in mood.

While we report multiple potential benefits of meditation training, our study is not without limitations. First, our active control group involved listening to music for the same duration as the meditation group; some participants in the music group subjectively reported that they used relaxation techniques similar to those used by the meditation group, such as relaxing imagery and using the breath as an anchor for their attention. This occurred despite the fact that the music group participants were not instructed to use any particular technique, and just to use their time to relax in any way they wished. Second, for our meditation group, some participants reported falling asleep during the meditation sessions indicating that they were not actively meditating during that time. Combined, this suggests our active control group may have reaped some of the same benefits as the meditation group and therefore we may have a conservative estimate of the potential for meditation to benefit attention and executive function in older adults; future research may wish to explore alternate control groups, such as a no-contact control or other control activities such as reading or watching TV. Finally, as this is a proof-of-concept study with the purpose of examining preliminary effects, we had a limited sample size and set our alpha at a liberal threshold of 0.10. We note that this sample size is smaller than what was shown in our original power analysis to find between-group differences at the *p* < 0.05 level. Further, given our sample size, we were unable to examine differences between sub-groups, such as how meditation effects may differ according to age, gender, and sex. However, we note that our effect sizes all indicate medium-to-large effects which provides promising evidence of the effects of meditation training on attention. Nevertheless, rigorous RCTs with stricter statistical thresholds are needed to validate the effects of meditation on attention. Additionally, future RCTs with large sample sizes to allow for sub-group analyses, different control groups, and a longer follow up period will allow for further verification of the effects reported in our pilot study.

In conclusion, 4 weeks of meditation training improved attention in healthy community-dwelling older adults. Attention is a critical cognitive function that supports mobility, executive functions, and performing activities of daily living; thus, improving attention in those who are at-risk for decline may help promote functional independence and quality of life in these individuals. With the focus of our study on laboratory-based measures, it would be worthwhile to examine how such results may translate to real-world tasks and functions, and to also examine the clinical significance of observed changes. Based on this a proof-of-concept study, a full-scale definitive trial with conservative significance thresholds and clinically meaningful endpoints (e.g., number of falls) should be conducted. Overall, we suggest that meditation may offer a cost-effective and scalable solution for boosting attention in older adults. Future research with larger sample sizes, stricter statistical thresholds, and post-intervention follow-up periods are warranted to examine the long-term benefits of meditation training and transferability to real-world outcomes.

## Data Availability

The raw data supporting the conclusion of this article will be made available by the authors, without undue reservation.
