# Supplementary material for: Four weeks of meditation training improves sustained attention in community-dwelling older adults: a proof-of-concept randomized controlled trial
Source: Front Aging. 2024 Mar 1;5:1322705. doi: 10.3389/fragi.2024.1322705 (PMC10940317; doi:10.3389/fragi.2024.1322705)
Supplement: Supplementary file 1 [file Table1.docx]

**Supplementary Table 1**

*Repeated measures ANOVA results with effect sizes*

| **Source** | **df** | **Mean square** | **F** | ***p*** | **η_p_^2^** |
| --- | --- | --- | --- | --- | --- |
| *Go trials accuracy* |  |  |  |  |  |
| Time | 1 | 152.963 | 15.752 | <0.001* | 0.344 |
| Time x group | 1 | 27.557 | 2.838 | 0.102* | 0.086 |
| Error | 30 |  |  |  |  |
| *Go trials reaction time* |  |  |  |  |  |
| Time | 1 | 0.547 | 0.242 | 0.626 | 0.008 |
| Time x group | 1 | 12.647 | 5.599 | 0.025* | 0.157 |
| Error | 30 |  |  |  |  |
| *No-go trials accuracy* |  |  |  |  |  |
| Time | 1 | 0.001 | 0.429 | 0.517 | 0.014 |
| Time x group | 1 | 0.004 | 1.991 | 0.169 | 0.062 |
| Error | 30 |  |  |  |  |
| *N2 component mean amplitude go trials* |  |  |  |  |  |
| Time | 1 | 0.002 | 0.000 | 0.991 | 0.000 |
| Time x group | 1 | 23.490 | 1.847 | 0.184 | 0.058 |
| Error | 30 |  |  |  |  |
| *N2 component peak latency go trials* |  |  |  |  |  |
| Time | 1 | 4503.452 | 3.652 | 0.066* | 0.109 |
| Time x group | 1 | 2507.452 | 2.033 | 0.164 | 0.063 |
| Error | 30 |  |  |  |  |
| *N2 component mean amplitude no-go trials* |  |  |  |  |  |
| Time | 1 | 5.688 | 0.372 | 0.546 | 0.012 |
| Time x group | 1 | 49.178 | 3.221 | 0.083* | 0.097 |
| Error | 30 |  |  |  |  |
| *N2 component peak latency no-go trials* |  |  |  |  |  |
| Time | 1 | 2238.354 | 1.541 | 0.224 | 0.049 |
| Time x group | 1 | 4400.041 | 3.029 | 0.092* | 0.092 |
| Error | 30 |  |  |  |  |
| *P3 component mean amplitude go trials* |  |  |  |  |  |
| Time | 1 | 106.647 | 3.347 | 0.077* | 0.100 |
| Time x group | 1 | 81.619 | 2.561 | 0.120 | 0.079 |
| Error | 30 |  |  |  |  |
| *P3 component peak latency go trials* |  |  |  |  |  |
| Time | 1 | 17164.655 | 3.123 | 0.087* | 0.094 |
| Time x group | 1 | 9630.905 | 1.752 | 0.196 | 0.055 |
| Error | 30 |  |  |  |  |
| *P3 component mean amplitude no-go trials* |  |  |  |  |  |
| Time | 1 | 38.704 | 0.617 | 0.438 | 0.020 |
| Time x group | 1 | 224.666 | 3.583 | 0.068* | 0.107 |
| Error | 30 |  |  |  |  |
| *P3 component peak latency no-go trials* |  |  |  |  |  |
| Time | 1 | 77.965 | 0.022 | 0.883 | 0.001 |
| Time x group | 1 | 207.902 | 0.058 | 0.811 | 0.002 |
| Error | 30 |  |  |  |  |
| *Digit span* |  |  |  |  |  |
| Time | 1 | 2.704 | 1.148 | 0.291 | 0.031 |
| Time x group | 1 | 12.809 | 5.437 | 0.025* | 0.131 |
| Error | 36 |  |  |  |  |
| *Trail Making test* |  |  |  |  |  |
| Time | 1 | 300.925 | 2.131 | 0.153 | 0.057 |
| Time x group | 1 | 138.216 | 0.979 | 0.329 | 0.027 |
| Error | 35 |  |  |  |  |
| *Stroop colour word test* |  |  |  |  |  |
| Time | 1 | 2.830 | 0.012 | 0.915 | 0.000 |
| Time x group | 1 | 0.361 | 0.001 | 0.969 | 0.000 |
| Error | 36 |  |  |  |  |
| *Activities balance confidence scale* |  |  |  |  |  |
| Time | 1 | 59.532 | 6.640 | 0.014* | 0.156 |
| Time x group | 1 | 0.525 | 0.059 | 0.810 | 0.002 |
| Error | 36 |  |  |  |  |
| *Timed up and go test* |  |  |  |  |  |
| Time | 1 | 7.473 | 6.829 | 0.013* | 0.159 |
| Time x group | 1 | 0.530 | 0.484 | 0.491 | 0.013 |
| Error | 36 |  |  |  |  |
| *Short physical performance battery* |  |  |  |  |  |
| Time | 1 | 0.378 | 0.322 | 0.574 | 0.010 |
| Time x group | 1 | 0.378 | 0.322 | 0.574 | 0.010 |
| Error | 33 |  |  |  |  |
| *DASS-21 Depression* |  |  |  |  |  |
| Time | 1 | 12.042 | 1.524 | 0.225 | 0.041 |
| Time x group | 1 | 2.358 | 0.298 | 0.588 | 0.008 |
| Error | 36 |  |  |  |  |
| *DASS-21 Anxiety* |  |  |  |  |  |
| Time | 1 | 51.064 | 4.828 | 0.035* | 0.118 |
| Time x group | 1 | 12.380 | 1.171 | 0.286 | 0.031 |
| Error | 36 |  |  |  |  |
| *DASS-21 Stress* |  |  |  |  |  |
| Time | 1 | 67.602 | 6.172 | 0.018* | 0.146 |
| Time x group | 1 | 7.076 | 0.646 | 0.427 | 0.018 |
| Error | 36 |  |  |  |  |
| *Cognitive failures questionnaire* |  |  |  |  |  |
| Time | 1 | 42.632 | 1.390 | 0.246 | 0.037 |
| Time x group | 1 | 118.421 | 3.862 | 0.057* | 0.097 |
| Error | 36 |  |  |  |  |

**p* < 0.100.
